# Effects of resistant dextrin for weight loss in overweight adults: a systematic review with a meta-analysis of randomized controlled trials

**DOI:** 10.1186/s40780-017-0084-9

**Published:** 2017-05-16

**Authors:** Junichi Mukai, Yuta Tsuge, Michiko Yamada, Katsuya Otori, Koichiro Atsuda

**Affiliations:** 1 Laboratory of Clinical Pharmacy Education, School of Pharmacy, Kitasato University, 5-9-1, Shirokane, Minato-ku, Tokyo, 108-8641 Japan; 2 Laboratory of Pharmacy Practice and Science I, 1-15-1, Kitasato, Minami-Ku, Sagamihara, Kanagawa 252-0375 Japan; 3 Department of Pharmacy, Kitasato University Medical Center, 6-100 Arai, Kitamoto-shi, Saitama 364-8501 Japan; 4 Department of Pharmacy, Kitasato University Hospital, 1-15-1, Kitasato, Minami-Ku, Sagamihara, Kanagawa 252-0375 Japan

**Keywords:** Dietary fiber, Resistant dextrin, Overweight, Meta-analysis, Systematic review

## Abstract

**Background:**

Randomized controlled trials (RCTs) reported that resistant dextrin (RD) exerted pleiotropic effects on humans. However, limited information is available on the effects of RD for weight loss. We conducted a systematic review with a meta-analysis to summarize the available literature and compare the efficacy of RD for weight loss with that of a placebo in overweight adults.

**Methods:**

We searched the electronic databases MEDLINE, EMBASE, The Cochrane Central Register of Controlled Trials (CENTRAL), CINAHL, Web of Science, ClincalTrials.gov, and Japana Centra Revuo Medicina (Ichushi-web) for studies from their onset to November 2016, and there was no language restriction. Trials were included if they were RCTs (1) comparing the effects of RD with a placebo in adults (18 years or older), (2) reporting body mass index, and (3) including overweight/obese subjects as defined by the authors of RCTs. The weighted mean difference with a 95% confidence interval (CI) was calculated using a random-effects model.

**Results:**

Of the 484 studies retrieved, 3 RCTs involving 275 subjects were included in our review. The durations of RCTs ranged between 8 and 12 weeks. All RCTs were conducted in Asian countries. RD significantly improved body mass index [mean difference −0.39 (95% CI −0.57 to −0.21) kg/m^2^, *p* < 0.01] and body weight [mean difference −0.81 (95% CI −0.93 to −0.69) kg, *p* < 0.01] in overweight adults.

**Conclusion:**

Our review suggests that RD exerts beneficial effects for weight loss in overweight adults. More RCTs with different populations and longer follow-ups are needed in order to confirm that supplementation with RD has beneficial effects for weight loss in overweight adults. We consider this review to provide important information for the future submission of food with health claims.

## Background

According to the World Health Organization, the prevalence of obesity has more than doubled worldwide in the past 30 years. In 2014, there were more than 1.9 billion overweight adults and 600 million obese adults [1]. The prevalence of obesity has increased in Asia, varying from 10% in India to 28.3% in Thailand. In Asia, the prevalence of diabetes is higher than that expected based on the prevalence of obesity [2, 3]. Dietary fiber has been reported to exert several beneficial effects. For example, a cross-over study reported that a high dietary fiber intake improved plasma glucose levels in patients with type 2 diabetes [4]. A cross-sectional study demonstrated that fiber intake was associated with weight loss [5]. Another clinical study showed that dietary fiber assisted with weight loss in overweight subjects [6]. Resistant dextrin (RD), a soluble dietary fiber, is an indigestible glucose polysaccharide (rich in α-1,2 or α-1,3 linkages) that is formed when starch is heated and treated with enzymes, and is made of wheat or maize starch. RD acts as a fermentation substrate in the colon [7, 8]. It has been classified as FOSHU (foods for special health uses) in Japan or GRAS (generally recognized as safe) by the Food and Drug Administration in the United States. Several studies have reported that RD exerts pleiotropic effects. For example, a recent randomized controlled trial (RCT) including healthy subjects found that RD significantly improved serum triglyceride levels and visceral fat accumulation over those with a placebo in a 12-week follow-up [9]. Another RCT that included overweight subjects demonstrated that body mass index (BMI) was significantly lower with RD than with a placebo [10]. Although a recent meta-analysis of 37 cross-over RCTs has revealed the attenuation of postprandial blood glucose in healthy subjects administered RD [11], to the best of our knowledge, there is only systematic review of RD with a focus on glycemic control. We hypothesized that the effects of RD for weight loss may be consistent across RCTs related to this area [10]. These findings may provide a novel insight into the effects of RD. Accordingly, we conducted a systematic review with a meta-analysis to summarize the available literature and compare the effects of RD for weight loss with those of a placebo in overweight adults.

## Methods

### Search methods for the identification of RCTs

We searched the electronic databases MEDLINE, EMBASE, The Cochrane Central Register of Controlled Trials (CENTRAL), CINAHL, Web of Science, ClincalTrials.gov, and Japana Centra Revuo Medicina (Ichushi-web) for studies from their onset to November 2016 using the following Medical Subject Headings and text words as search terms: “dextrins”, “maltodextrin”, “resistant dextrin”, “resistant maltodextrin”, “indigestible dextrin”, “indigestible maltodextrin”, “nutriose”, “randomized”, and “randomized control trial”. We used a filter to restrict our search to “Randomized Controlled Trial” when using MEDLINE. The search strategy for MEDLINE was as follows: “Dextrins”[Mesh] OR “maltodextrin” [Supplementary Concept] OR “NUTRIOSE” [Supplementary Concept]. A reference search was also implemented from relevant studies in order to identify more RCTs. There was no language restriction. Trials were included if they were RCTs (1) comparing the effects of RD with a placebo in adults (18 years or older), (2) reporting BMI, and (3) including overweight/obese subjects as defined by the authors of RCTs. We excluded RCTs involving healthy subjects. We also excluded cross-over trials. The study search was undertaken independently by two authors (MY and JM). Any discrepancies were settled by discussions between the two assessors. They also assessed RCT quality. We extracted data on the trial country, trial design, daily dose of RD, subjects (mean BMI, Hemoglobin A1c, and age at baseline), duration of the intervention, trial population, reporting of BMI, body weight, and adverse events. BMI was used as the primary endpoint. The secondary endpoint was body weight. This systematic review did not require Ethics Committee approval.

### Quality assessment

Study quality was quantified by the Jadad scale, which is used to evaluate the appropriateness of the randomization technique, the method used for double-masking, and descriptions of dropouts or withdrawals [12]. The Jadad scale ranges between zero and five. Studies that scored 3 points or higher were defined as high quality and were included in the analysis. Additionally, the risk of bias of the RCTs included was assessed based on the Cochrane handbook of systematic reviews [13]. Seven items were examined for the risk of bias: random sequence generation, allocation concealment, the blinding of participants and personnel, blinding of outcome assessments, incomplete outcome data, free of selective reporting, and baseline imbalance as other sources of bias. Each of the seven items was scored as a “low risk”, “unclear risk”, or “high risk”.

### Statistical analysis

The weighted mean difference with a 95% confidence interval (CI) was calculated for each outcome. The heterogeneity of each outcome was evaluated using chi-squared and I^2^ statistics. A value of 50% or more was defined to represent marked heterogeneity according to the Cochrane handbook of systematic reviews [13]. We used a random-effects model (the DerSimonian and Laird method [14]) to more conservatively assess outcomes. In the meta-analysis, multiple RD groups from a trial were combined into a single group [13]. A sensitivity analysis was performed using a fixed-effects model (the inverse variance method [13]). Additionally, subgroup analyses were performed by excluding patients with diabetes, subjects with different BMIs (>25 kg/m^2^), and RCTs with the largest sample sizes. If necessary, variances for the change from baseline were calculated using a correlation efficient of 0.5 [15]. The meta-analysis was performed using review manager 5.3 software (Cochrane Collaboration, Oxford, UK). A P value less than 0.05 was considered to be significant.

## Results

**Fig. 1:**
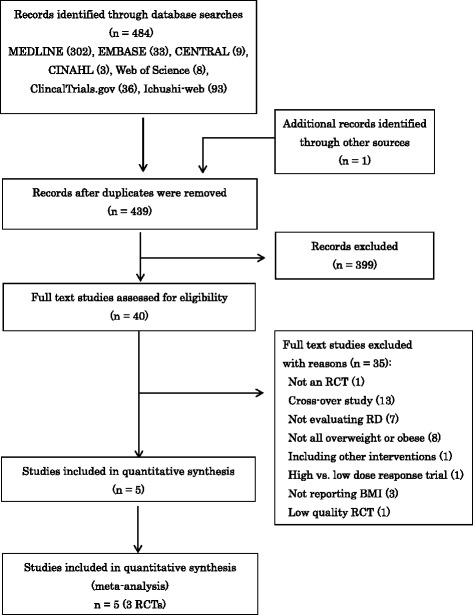
Identification process for eligible randomized controlled trials (RCTs)

We identified 484 studies in the database search. Forty full text studies were retrieved after screening titles and abstracts. Five studies involving 3 RCTs were ultimately included in our review. Figure 1 shows the identification process for eligible RCTs [10, 16–19] following PRISMA [20]. Table 1 shows the characteristics of RCTs included in the meta-analysis. Two trials conducted by Guerin et al. [16, 17] were the same RCT (Guerin 2013) with multiple reports, except for adverse events. Similarly, the trials conducted by Li et al. [18] and Guerin et al. [19] were the same RCT (Li 2010) with different main outcomes (i.e., BMI and body weight). All trials were randomized, placebo-controlled, double-masked studies. The sample sizes of the RCTs ranged between 55 and 113 subjects. The doses of RD ranged between 10 and 34 mg/day. The durations of RCTs ranged between 8 and 12 weeks. Only one trial enrolled participants with type 2 diabetes [10]. All RCTs were conducted in Asian countries such as Iran and China. All trials were published in English.

**Table 1:** Characteristics of RCTs included in the meta-analysis

Trial (Year)	Country	Design	Intervention (N)	Follow-up (weeks)	Co-intervention	Jadad score	Baseline BMI (kg/m^2^)	Baseline HbA1c (%)	Age (year)	Male (%)	Trial population	Adverse events (%)
Aliasgharzadeh (2015) [10]	Iran	D	RD 5 g with a cup of water during a meal twice daily (30), or maltodextrin with a cup of water (25)	8	OADs	5	RD: 31.8 Control: 30.8	RD: 7.8 Control: 8.2	RD: 49.2 Control: 49.6	0	T2DM BMI > 25	None
Guerin (2013) [16, 17]	China	D	250 ml of orange juice containing RD 4 g (20), RD 7 g (20), RD 9 g (20), or RD 12 g twice daily (20), or 250 ml of orange juice only (20)	9	None	4	RD: 26.0 Control: 26.0	N. R.	RD: 44.7 Control: 45.0	50	BMI 24–28	None
Li (2010) [18, 19]	China	D	250 ml of fruit juice containing 17 g RD twice daily (57), or 250 ml of fruit juice containing maltodextrin (56)	12	None	5	RD: 24.5 Control: 24.5	RD: 5.8 Control: 5.8	RD: 30.4 Control: 31.6	100	BMI 24–28	None

*RCT* randomized controlled trial, *D* double masked, *RD* resistant dextrin, *N. R.* not reported, *OADs* oral antidiabetic drugs, *BMI* body mass index, *HbA1c* Hemoglobin A1c, *T2DM* type 2 diabetes mellitus

### Quality assessment

All RCTs were assessed as high quality (Table 1). One RCT that scored 4 points provided no information on participant withdrawal [16, 17]. We also assessed the risk of bias of RCTs based on the Cochrane handbook [13]. Three trials had a low risk of bias for all seven domains [10, 16–19].

### Meta-analysis of a comparison of weight loss between RD and the control

**Fig. 2:**
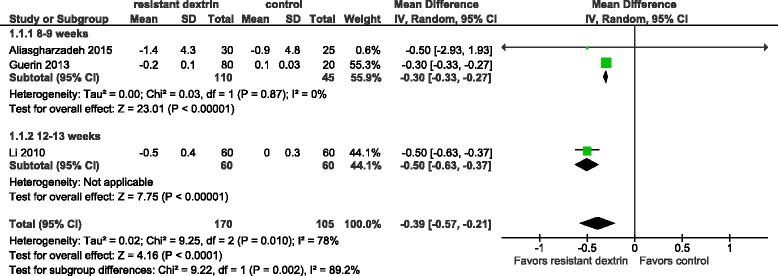
Meta-analysis of a comparison of BMI between RD and the control

Three trials were included in the meta-analysis of a comparison of BMI between RD and the control [10, 16–19]. Statistical heterogeneity was observed among trials (I^2^ = 78%). RD significantly decreased BMI in overweight adults [mean difference −0.39 (95% CI −0.57 to −0.21) kg/m^2^, *p* < 0.01]. The subgroup analysis by follow-up at 8–9 weeks and 12–13 weeks showed a significant result [mean difference −0.30 (95% CI −0.33 to −0.27) kg/m^2^, *p* < 0.01; mean difference −0.50 (95% CI −0.63 to −0.21) kg/m^2^, *p* < 0.01] (Fig. 2).

**Fig. 3:**
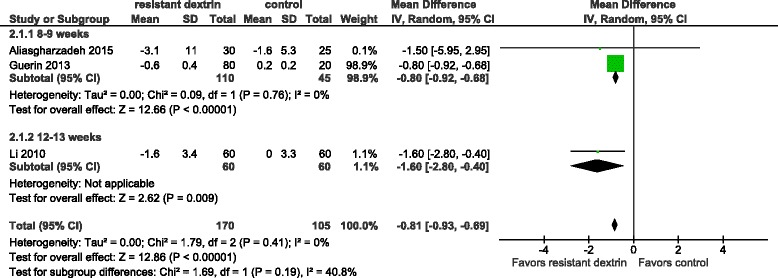
Meta-analysis of a comparison of body weight between RD and the control

Three trials [10, 16–19] were included in the meta-analysis of a comparison of body weight between RD and the control. No statistical heterogeneity was observed across trials (I^2^ = 0%). RD significantly reduced body weight in overweight adults [mean difference −0.81 (95% CI −0.93 to −0.69) kg, *p* < 0.01]. The subgroup analysis by follow-up at 8–9 weeks and 12–13 weeks showed a significant result [mean difference −0.80 (95% CI −0.92 to −0.68) kg, *p* < 0.01; mean difference −1.60 (95% CI −2.80 to −0.40) kg, *p* < 0.01] (Fig. 3).

### Adverse events

None of the RCTs examined reported any adverse events (Table 1).

### Additional analyses

The subgroup analysis excluding patients with type 2 diabetes [10] showed that the effects of RD on BMI and body weight remained unchanged (Table 2). Another subgroup analysis including subjects with a BMI of 25 or more showed no significant results in BMI or body weight. Another subgroup analysis excluding the RCT with the largest sample size (Li 2010) showed significant results in BMI and body weight [18, 19] (Table 2). The results of the sensitivity analysis using a fixed-effects model were similar to those obtained using a random-effects model (Table 2).

**Table 2:** Results of sub-analyses

	Outcome	Trial, *n*	RD, *n*	Control, *n*	Mean Difference [95% CI]	Heterogeneity (%)	Test for the overall effect (*P* value)
Excluding patients with type 2 diabetes	BMI (kg/m^2^)	2	140	80	−0.38 [−0.60, −0.15]	92	0.001
	BW (kg)	2	140	80	−0.97 [−1.61,-0.33]	41	0.003
Including subjects with a BMI > 25	BMI (kg/m^2^)	1	30	25	−0.50 [−2.93,1.93]	N. A.	0.69
	BW (kg)	1	30	25	−1.50 [−5.95, 2.95]	N. A.	0.51
Excluding the RCT with the largest sample size (Li 2010)	BMI (kg/m^2^)	2	110	45	−0.30 [−0.33, −0.27]	0	<0.001
	BW (kg)	2	110	45	−0.80 [−0.92, −0.68]	0	<0.001
Sensitivity analysis using the fixed-effect model	BMI (kg/m^2^)	3	170	105	−0.31 [−0.33, −0.28]	78	<0.001
	BW (kg)	3	170	105	−0.81 [−0.93, −0.69]	0	<0.001

*RD* resistant dextrin, *RCT* randomized controlled trial, *BMI* body mass index, *BW* body weight, *N. A.* not applicable

## Discussion

To the best of our knowledge, we are the first to conduct a systematic review with a meta-analysis in order to assess the effects of RD for weight loss with those of a placebo in overweight adults. Our review suggests that RD exerts beneficial effects on BMI and body weight in overweight adults.

With a focus on each individual RCT, it is noteworthy that the studies by Li in 2010 [18, 19] had the lowest mean BMI at baseline (24.5 kg/m^2^) and the highest daily dose of RD (34 g/day) in our review. Additionally, the RCT showed the greatest mean differences in BMI and body weight, which were significant [mean difference −0.5 (95% CI −0.6 to −0.4) kg/m^2^, mean difference −1.6 (95% CI −2.8 to −0.4) kg/m^2^, respectively]. In contrast, another study did not obtain significant findings in any case [10]. The trial by Aliasgharzadeh et al. [10] also had the greatest mean difference in BMI, but this was not significant [mean difference −0.5 (95% CI −2.9 to 1.9) kg/m^2^]; however, patients in that trial [10] took glibenclamide, which has been suggested to affect weight gain [21].

As expected, when we excluded the RCT with the largest sample size (Li 2010) [18, 19], the effects of BMI and body weight remained significant between RD and the control. In other analyses, excluding the trial by Aliasgharzadeh et al. [10], which used oral antidiabetic drugs in type 2 diabetic patients, significant differences were observed in the combined results of BMI and body weight. We speculated that the trial with the greatest weight (Guerin 2013) [16, 17] given by the smallest standard deviation may contribute to these significant results.

The Food and Drug Administration assessment indicated that the proportion of patients who achieved clinically meaningful weight loss of 5% at one year by using an anti-obesity drug was 50% or less [22, 23]. Another RCT showed that the initial weight loss response at 12 weeks predicted weight loss after one year [24]. If RD achieves an initial body weight loss of 5% at least at one year, it may be of clinical value for weight loss. However, our review only included 3 RCTs with a duration of 12 weeks or shorter. Therefore, a larger number of RCTs with a longer duration are needed in order to evaluate the efficacy of RD.

Our results showed that BMI was significantly lower with RD than with the control. However, all RCTs pooled in our review were conducted in Asian countries. Additionally, two RCTs included Chinese participants who had a lower mean BMI of 30 or less (Table 1). Collectively, this means that RD may only improve BMI in East-Asians with a BMI of 30 or less. A narrative review showed that BMI was lower in Asians than in Non-Asians such Europeans. For example, the prevalence of obesity with a BMI of 30 or more is approximately seven-fold higher among Europeans than Chinese [2]. Therefore, the efficacy of RD for weight loss in Non-Asians or Asians with a BMI of 30 or more remains unclear.

A recent meta-analysis revealed that dietary fiber such as RD significantly increased self-reported feelings of satiety in healthy adults [25]. Aliasgharzadeh et al. [10] hypothesized that RD may enhance satiety by stimulating the secretion of gut hormones such as glucagon-like peptide-1, similar to other dietary fibers [26]. Furthermore, an epidemiological study demonstrated that fat correlated with increases in endotoxin [27]. These mechanisms appear to be involved in RD-induced reductions in BMI and body weight.

Our review has some limitations. There may be publication biases because this review only included published RCTs. In addition, we did not analyze the publication bias because the number of RCTs that we were able to collect was too small (n = 3) to test the funnel plot. Therefore, the present results may be affected if unpublished findings become available. The combined results need to be interpreted with caution because there was significant heterogeneity in at least one outcome. The small number of RCTs in our review may cause this heterogeneity.

## Conclusion

Based on the limited evidence available, our review suggests that RD exerts beneficial effects on BMI and body weight in overweight adults. However, our results represent considerable heterogeneity and the trials pooled in our review have shorter follow-ups or limited populations. Thus, more RCTs with different populations, or longer follow-ups are needed in order to confirm that supplementation with RD has beneficial effects for weight loss in overweight adults. We consider this review to provide important information for the future submission of food with health claims.

## Abbreviations

BMI: Body mass index
CI: Confidence interval
RCT: Randomized controlled trial
RD: Resistant dextrin
